# A randomised controlled trial of non-invasive ventilation compared with extracorporeal carbon dioxide removal for acute hypercapnic exacerbations of chronic obstructive pulmonary disease

**DOI:** 10.1186/s13613-022-01006-8

**Published:** 2022-04-21

**Authors:** Nicholas A. Barrett, Nicholas Hart, Kathleen J. R. Daly, Martina Marotti, Eirini Kostakou, Chris Carlin, Stephanie Lua, Suveer Singh, Andrew Bentley, Abdel Douiri, Luigi Camporota

**Affiliations:** 1 Department of Critical Care, NHS Foundation Trust, Guy’s and St ThomasWestminster Bridge Rd, London, SE1 7EH UK; 2 Centre for Human & Applied Physiological Sciences (CHAPS), School of Basic & Medical Biosciences, Faculty of Life Sciences & Medicine, King’s College London, London, WC2R 2LS UK; 3 Lane Fox Respiratory Unit, Guy’s and St. Thomas’ NHS Foundation Trust, Westminster Bridge Rd, London, SE1 7EH UK; 4 Dept. of Respiratory Medicine, Queen Elizabeth University Hospital, Glasgow, G51 4TF UK; 5 Department of Respiratory and Critical Care Medicine, Chelsea & Westminster Hospital, London, SW10 9NH UK; 6 Department of Intensive Care & Respiratory Medicine, Wythenshawe Hospital, Manchester University NHS Foundation Trust, Manchester, M23 9LT UK; 7 School of Population Health & Environmental Sciences, King’s College London, London, WC2R 2LS UK; 8 National Institute for Health Research Biomedical Research Centre, Guy’s and St. Thomas’ NHS Trust and King’s College London, London, WC2R 2LS UK

**Keywords:** Acute exacerbations of chronic obstructive pulmonary disease, AECOPD, Extracorporeal CO_2_ removal, ECCO_2_R, Non-invasive ventilation, NIV

## Abstract

**Background:**

Patients presenting with acute hypercapnic respiratory failure due to exacerbations of chronic obstructive pulmonary disease (AECOPD) are typically managed with non-invasive ventilation (NIV). The impact of low-flow extracorporeal carbon dioxide removal (ECCO_2_R) on outcome in these patients has not been explored in randomised trials.

**Methods:**

Open-label randomised trial comparing NIV (NIV arm) with ECCO_2_R (ECCO_2_R arm) in patients with AECOPD at high risk of NIV failure (pH < 7.30 after ≥ 1 h of NIV). The primary endpoint was time to cessation of NIV. Secondary outcomes included device tolerance and complications, changes in arterial blood gases, hospital survival.

**Results:**

Eighteen patients (median age 67.5, IQR (61.5–71) years; median GOLD stage 3 were enrolled (nine in each arm). Time to NIV discontinuation was shorter with ECCO_2_R (7:00 (6:18–8:30) vs 24:30 (18:15–49:45) h, *p* = 0.004). Arterial pH was higher with ECCO_2_R at 4 h post-randomisation (7.35 (7.31–7.37) vs 7.25 (7.21–7.26), *p* < 0.001). Partial pressure of arterial CO_2_ (PaCO_2_) was significantly lower with ECCO_2_R at 4 h (6.8 (6.2–7.15) vs 8.3 (7.74–9.3) kPa; *p* = 0.024). Dyspnoea and comfort both rapidly improved with commencement of ECCO_2_R. There were no severe or life-threatening complications in the study population. There were no episodes of major bleeding or red blood cell transfusion in either group. ICU and hospital length of stay were longer with ECCO_2_R, and there was no difference in 90-day mortality or functional outcomes at follow-up.

**Interpretation:**

There is evidence of benefit associated with ECCO_2_R with time to improvement in respiratory acidosis, in respiratory physiology and an immediate improvement in patient comfort and dyspnoea with commencement of ECCO_2_R. In addition, there was minimal clinically significant adverse events associated with ECCO_2_R use in patients with AECOPD at risk of failing or not tolerating NIV. However, the ICU and hospital lengths of stay were longer in the ECCO_2_R for similar outcomes.

*Trial *registration The trial is prospectively registered on ClinicalTrials.gov: NCT02086084. Registered on 13th March 2014, https://clinicaltrials.gov/ct2/show/NCT02086084?cond=ecco2r&draw=2&rank=8

**Supplementary Information:**

The online version contains supplementary material available at 10.1186/s13613-022-01006-8.

## Background

Chronic obstructive pulmonary disease (COPD) is characterized by progressive and not fully reversible expiratory airflow limitation with intermittent acute exacerbations (AECOPD) complicated by hypercapnic respiratory failure (arterial partial pressure of carbon dioxide (PaCO_2_) > 6.5 kPa (49 mmHg) and pH < 7.35) [1]. In these patients, non-invasive ventilation (NIV) decreases the rate of tracheal intubation [2] and provides a significant survival benefit [2]. However, 15–30% of patients on NIV experience treatment failure and receive invasive mechanical ventilation (IMV) [3]. Reasons for NIV treatment failure include device or mask intolerance, discomfort, or unresolving respiratory acidosis, tachypnoea and respiratory distress [4–6]. These patients are at significantly higher risk of death [7].

Extracorporeal carbon dioxide removal (ECCO_2_R) pumps venous blood through an extracorporeal circuit with a gas exchanging membrane to clear CO_2_ [8, 9]. ECCO_2_R has been shown to have physiological benefits in pre-clinical trials [10] and uncontrolled case series in AECOPD [11–13]. To date, there have been no randomised controlled trials on the role of ECCO_2_R in AECOPD. There are 6 further trials currently registered with clinicaltrials.gov [14].

The hypothesis for this trial is that ECCO_2_R results in faster correction of hypercapnia and earlier cessation of NIV, by at least 12 h. Time to cessation of NIV is an important outcome as longer duration of NIV is associated with greater complications and discomfort—both independent predictors of NIV failure [5, 6].

## Methods

This study was a randomised, open-label, parallel-arm trial comparing standard therapy using NIV (NIV arm) with ECCO_2_R added to NIV (ECCO_2_R arm) in adults with AECOPD. Patients were included if they were over 18 years of age, had a history of COPD presenting with AECOPD and with a persisting pH < 7.30 due to hypercapnia after initial medical therapy and at least 1 h of NIV. Patients were randomised following written informed consent by the patient or nominated legal representative. Randomisation was computer-generated and allocation was concealed in opaque, sealed envelopes. Patients were excluded if they had acute multiple organ failure, intolerance, allergy or contraindication to heparin or a contraindication to NIV.

Patients were randomised to continuation of NIV alone or to the addition of ECCO_2_R to NIV. The full trial methodology has been published [15] (Additional file 1: Appendix S1 and Additional file 2: Figure S1). NIV was delivered using an ICU ventilator in NIV mode (Draeger V500, Germany) with a mask specifically designed for dual limb ventilators (Freemotion, Fisher and Paykel, New Zealand). ECCO_2_R was delivered using the Hemolung Respiratory Assist System (ALung Technologies, USA). The device has a cross-sectional membrane area of 0.59m^2^ and has an extracorporeal blood flow between 300 and 500 mL/min. Cannulation was with a dual lumen cannula inserted in either femoral or jugular veins using previously published methods [11]. Membrane VCO_2_ reported by the device was recorded. This has been previously shown to be consistent with that calculated using trans-membrane blood gases [16]. ECCO_2_R and heparin were managed in accordance with agreed institutional protocols (Additional file 1: Appendix S1). ECCO_2_R was weaned as the respiratory failure improved, with a goal of maintaining a respiratory rate of 25 or less and a pH 7.35–7.45. Once the sweep gas flow was reduced to 1L/minute for at least 4 h, the sweep gas was discontinued for 4–12 h. If there were no signs of respiratory failure at this point then the ECCO_2_R device was stopped and the cannula removed.

The primary outcome was time to discontinuation of NIV. Time to cessation of NIV was based on a combination of patient preference and physiological indicators—improvement in respiratory rate to less than 25 and pH more than 7.35. Short breaks for meals or patient comfort were allowed and did not count as discontinuing NIV. It was estimated that the addition of ECCO_2_R would reduce NIV duration by at least 12 h. When patients in the NIV arm had ceased NIV they were transferred to the ward the same day. The estimated sample size—1:1 enrolment ratio—was 12 patients in each arm. This would achieve 80% power to reject the null hypothesis of equal means when the population mean difference is 12 h with a standard deviation of 10 h with alpha level of 5% and a loss to follow-up of 10%. The trial was ceased early due to the onset of the SARS-2 Coronavirus pandemic resulting in all non-COVID-19-related research being ceased in the UK.

Secondary outcomes included physiological measurements, ICU and hospital length of stay (LOS) and outcomes (90-day mortality). Adverse outcomes included incidence of major haemorrhage (according to the ISTH bleeding score [17]), thrombosis, haemolysis, mechanical complications and need for IMV. Subjective discomfort and dyspnoea were measured using a visual analogue scale (VAS) (0–100 mm). A higher score indicates greater subjective discomfort or dyspnoea. Quality-of-life measurements, including the COPD assessment test (CAT) [18], the St George’s respiratory questionnaire (SGRQ) [19] and the EuroQuol-5D–5L [20] were administered at the 90-day follow-up visit. Recruitment was ceased by the investigators due to slow recruitment and with the onset of the SARS-CoV-2 pandemic leading to the cessation of all non-COVID research in critical care in the NHS.

### Ethical approval

The trial protocol was approved by the Cambridge NHS Human Research Authority Research Ethics Committee (14/EE/0109).

### Statistics

Statistical analysis was performed using Prism 9.1.1 for Mac (GraphPad, San Diego, USA). All data is presented as median (inter-quartile range). Data was tested for normal distribution using a Kolmogorov–Smirnov test and presented as median (inter-quartile range). Inter-group differences with continuous unpaired, non-parametric data were compared using a Mann–Whitney *U* test. Inter-group differences with discrete paired, non-parametric data were compared using a Wilcoxon matched pairs signed rank test. Intra-group differences over time with continuous, parametric data were compared using a one-way ANOVA with Dunnett’s correction for post-hoc comparisons. Intra-group differences over time with continuous non-parametric data were compared using Friedman’s test with Dunnett’s correction for post-hoc comparisons. Categorical data were compared using a Chi-squared analysis. Survival was analysed using a log-rank test. Statistical significance was defined as *p* < 0.05.

## Results

### Baseline characteristics

**Fig. 1:**
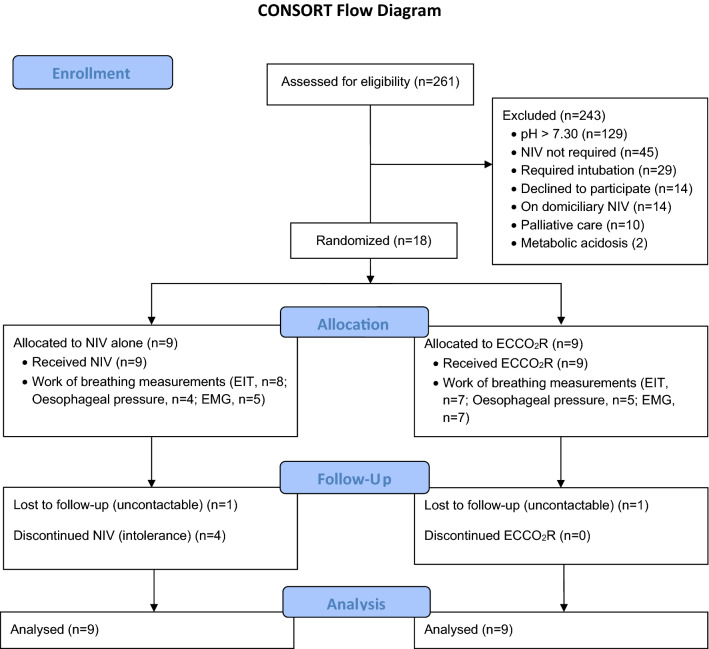
Consort diagram

Between December 2017 and March 2020, 261 potentially eligible patients were screened, 32 patients met inclusion criteria, 18 consented and were randomised (Fig. 1). Nine were randomised to each group (Table 1). All patients had severe COPD (median GOLD stage 3 in both groups), but no patients were receiving chronic domiciliary ventilation. Two were lost to follow-up, one from each group, and were considered alive for the analysis (data from the UK NHS database). Patients in both groups were comparable although baseline respiratory rate was higher with ECCO_2_R (24 [20–28] vs 29 [26–32] breaths/min, *p* < 0.05), haemoglobin was higher with ECCO_2_R (130 (120–136) vs 151(143–157) g/L *p* < 0.05), as was c-reactive protein (13 (3.5–16) vs 32 (30–51) mg/L, *p* < 0.05).

**Table 1:** Demographic data

	NIV	ECCO_2_R
Demographic data		
Age (years)	69 (61–71)	65 (63–71)
BMI	22.19 (21.72–30.9)	24.67 (23.78–26.99)
Sex (F)	3	5
FEV1 (L)	0.84 (0.59–1.1)	0.97 (0.7–1.32)
FEV1 (% predicted) (%)	38 (21–45)	39.8 (39–46)
FVC (L)	2.3 (1.34–2.6)	2.6 (1.7–3.3)
FVC (% predicted) (%)	63 (33–105)	82 (63–92)
FEV1/FVC	48 (32–49)	44 (37–48)
GOLD stage	3 (3–4)	3 (3–3)
Pack years smoked	40 (20–60)	40 (39–45)
Baseline observations		
Systolic blood pressure (mmHg)	120 (105–144)	130 (112–139)
Respiratory rate (breaths/min)	24 (20–28)	29 (26–32)* (* p* = 0.0371)
SpO_2_ (%)	91 (90–92)	91 (87–93)
Heart rate (beats/min)	100 (86–113)	109 (100–116)
Presenting arterial blood gas		
PaO_2_ (kPa)	8.67 (8.63–10.57)	7.33 (7.1–8.55)
PaCO_2_ (kPa)	9.18 (8.94–10.31)	9.75 (8.14–9.78)
pH	7.23 (7.23–7.27)	7.26 (7.25–7.28)
HCO_3_ (mmol/L)	31 (28.2–31.4)	29.5 (28.88–30.64)
Initial NIV settings		
EPAP (cmH_2_O)	5 (5–5)	6 (5–6)
IPAP (cmH_2_O)	18 (15–22)	18 (16–20)
FiO_2_ (%)	32 (26–40)	35 (28–40)
Arterial blood gas after 1 h NIV		
PaO_2_ (kPa)	8.37 (8.05–8.83)	8.89 (7.9–9.41)
PaCO_2_ (kPa)	9.16 (8.23–10.02)	9.34 (8.49–9.65)
pH	7.27 (7.24–7.27)	7.27 (7.25–7.27)
HCO_3_ (mmol/L)	29.1 (26.7–30.8)	27.9 (27.7–30.52)
Baseline laboratory investigations		
Leukocytes (× 10^9^/L)	8.9 (6.8–10.4)	9.1 (8.3–11.8)
Haemoglobin (g/L)	130 (120–136)	151 (143–157)* (* p* = 0.0411)
Platelets (× 10^9^/L)	251 (172–288)	204 (163–308)
Creatinine (umol/L)	99 (57–136)	77 (69–80)
Bilirubin (umol/L)	6 (4–6)	7 (5.5–12)
C-reactive protein (mg/L)	13 (3.5–16)	32 (30–51)* (* p* = 0.0199)

All data is presented as median (IQR) .* *p* < 0.05

### ECCO2R

All patients were cannulated via the femoral vein by patient choice as they preferred to not lie flat for jugular insertion. Blood and sweep flow rates were all within the operating range of the device (Additional file 3: Table S6). ECCO_2_R was ceased after a median (IQR) of 96 (60–138) h following successful weaning for all patients. CO_2_ clearance through the membrane lung (VCO_2ML_) was a median of 88 (83–104) mL/min in the first hour and was maintained during the first 48 h (Additional file 3: Table S6).

### Physiological changes post-randomisation

**Fig. 2:**
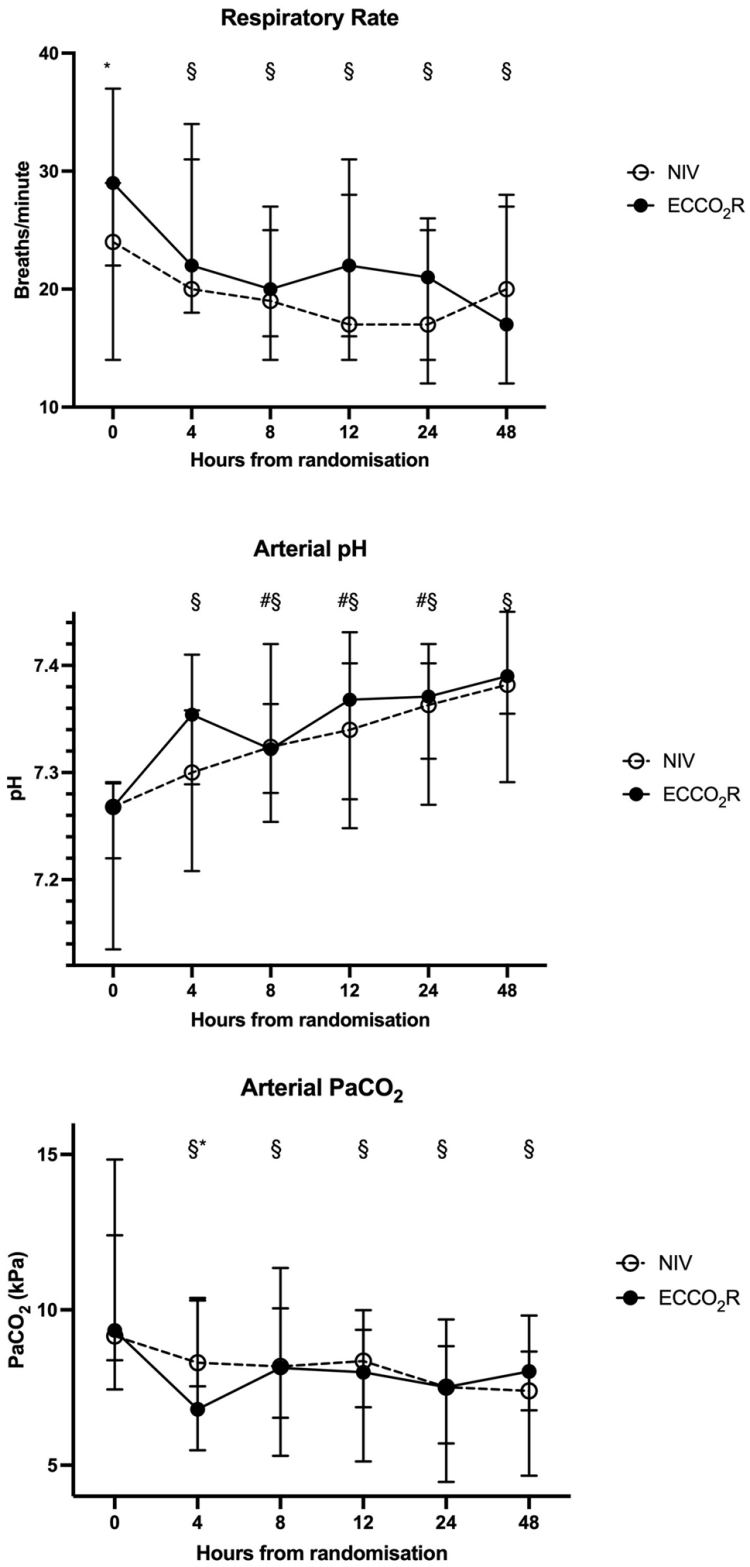
Respiratory rate, arterial pH and PaCO_2_ over the first 48 h. **a**: Respiratory rate over the first 48 h (* statistically significant difference between the NIV and ECCO_2_R groups (*p* < 0.05); ^ statistically significant difference over time in the ECCO_2_R group compared with baseline (time 0) (*p* < 0.05)); **b**: Arterial pH over the first 48 h (* statistically significant difference between the NIV and ECCO_2_R groups (*p* < 0.05); ^ statistically significant difference over time in the ECCO_2_R group compared with baseline (time 0) (*p* < 0.05); ^#^ statistically significant difference over time in the NIV group compared with baseline (time 0) (*p* < 0.05)); **c**: PaCO_2_ over the first 48 h (* statistically significant difference between the NIV and ECCO_2_R groups (*p* < 0.05); ^ statistically significant difference over time in the ECCO_2_R group compared with baseline (time 0) (*p* < 0.05); ^#^ statistically significant difference over time in the NIV group compared with baseline (time 0) (*p* < 0.05))

Levels of respiratory support did not differ between groups (Additional file 3: Table S1). The respiratory rate was higher with ECCO_2_R compared with NIV at baseline and 12 h post randomisation (22(20–24) vs 17 (15–19) breaths/min, *p* = 0.038) (Fig. 2a, Additional file 3: Table S1). There was no significant difference in respiratory rate over the first 48 h with NIV (Fig. 2a, Additional file 3: Table S1). There was, however, a significant reduction in respiratory rate with ECCO_2_R compared to baseline at four (22 (20–25) vs 29 (26–32) breaths/min, *p* = 0.039), eight (20 (20–22) vs 29 (26–32) breaths/min, *p* = 0.015), twelve (22 (20–24) vs 29 (26–32) breaths/min, *p* = 0.015), 24 (21 (20–23) vs 29 (26–32) breaths/min, *p* = 0.039) and 48 h (17 (16–23) vs 29 (26–32) breaths/min, *p* = 0.015).

Arterial pH was not significantly different between the two groups (Fig. 2b, Additional file 3: Table S1). With ECCO_2_R, the arterial pH in was significantly higher than baseline at each timepoint for the first 48 h (Fig. 2b, Additional file 3: Table S1). With NIV, the arterial pH was significantly higher than baseline at 8 h (7.32 (7.28–7.33) vs 7.27 (7.21–7.27), *p* = 0.022) and remained significantly higher at 12 and 24 h (Fig. 2b).

Partial pressure of arterial CO_2_ (PaCO_2_) was significantly lower with ECCO_2_R compared with NIV at 4 h (6.8 (6.2–7.15) vs 8.3 (7.74–9.3) kPa; *p* = 0.024) following randomisation (Fig. 2c). With ECCO_2_R, the arterial CO_2_ was significantly lower than baseline at each timepoint for the first 48 h. With NIV, the arterial CO_2_ was not statistically different to baseline at any timepoint in the first 48 h.

### Time on NIV

**Fig. 3.**
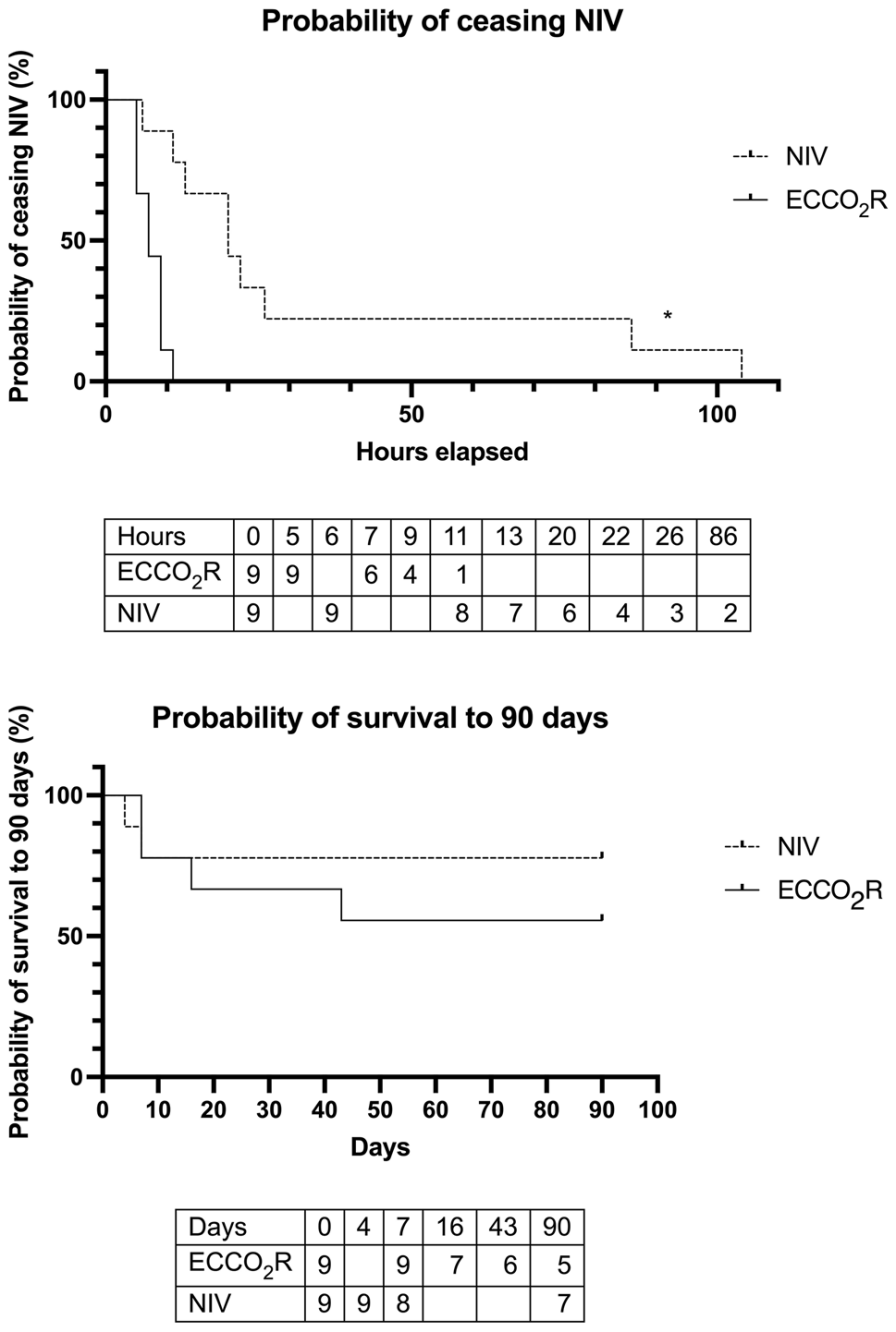
**a** Probability of remaining on NIV over time; **b** probability of survival to 90 days

Four patients in the NIV arm ceased NIV against the treating clinician’s advice. Median time from randomisation to cannulation and commencing ECCO_2_R was 2:27 (1:22–2:50) h (Table 2). Time from randomisation to pH > 7.35 was significantly lower with ECCO_2_R (5:32 (3:39–11:48) vs 23:58 (22:48–26:55) h, *p* = 0.024). Time to NIV discontinuation was significantly shorter with ECCO_2_R (7:00 (6:18–8:30) vs 24:30 (18:15–49:45) h, *p* = 0.004) (Table 2, Fig. 3a).

**Table 2:** Time to event following randomisation

	NIV	ECCO_2_R
First time pH > 7.35	15:22 (± 12:11)	10:57 (± 14:00)
NIV duration	37:28 (± 35:53)	7:15 (± 2:17)*
ECCO_2_R duration		100:05 (± 53:48)
First sat out of bed	21:53 (± 24:46)	63:06 (± 53:09)
First stand with assistance	33:40 (± 39:08)	59:46 (± 51:16)
First walk with assistance	44:27 (± 57:37)	91:08 (± 77:58)
First oral intake	31:19 (± 40:37)	24:38 (± 19:07)
ICU discharge	59:58 (± 37:54)	177:42 (± 87:10)*
Hospital discharge	160:16 (± 85:05)	276:04 (± 88:27)*

Mean (± SD) time is presented in the format hours:minutes (*p < 0.05)

### Subjective discomfort and dyspnoea

**Fig. 4:**
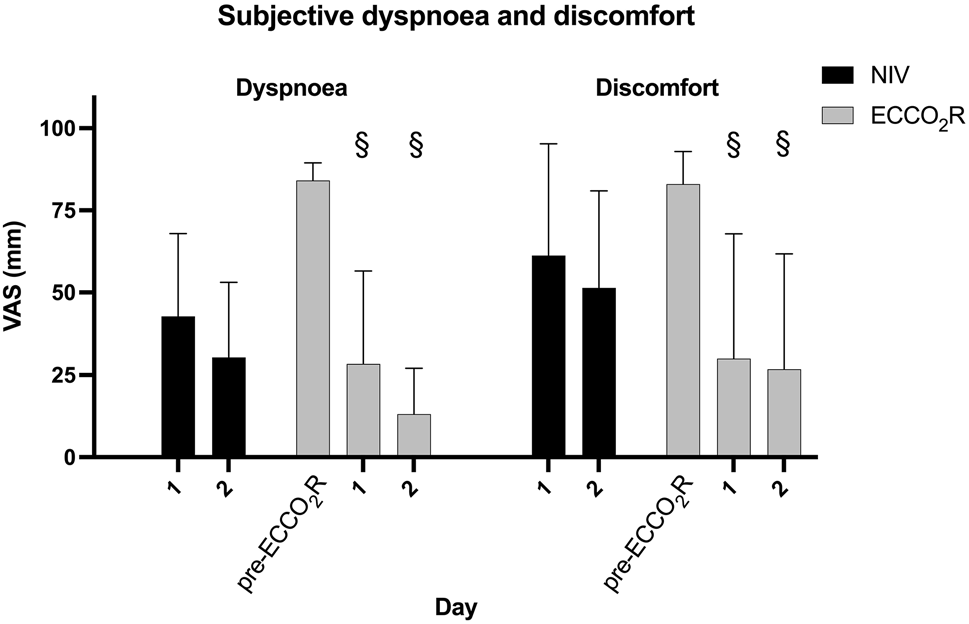
Change in dyspnoea and discomfort according to the visual analogue scale where 0 is least dyspnoeic/uncomfortable and 100 is most dyspnoeic/uncomfortable (§ *p*<0.05 difference between measurement and baseline within in the ECCO2R group)

ECCO_2_R resulted in a rapid and significant reduction in VAS for discomfort (84 (78–87) vs 13 (4–65), *p* = 0.0156) and dyspnoea (85 (80–87) vs 20 (7–52), *p* < 0.01). There were no significant differences between ECCO_2_R and NIV in either dyspnoea or discomfort at any timepoint. The discomfort and dyspnoea scores did not change between days 1 and 2 for NIV or ECCO_2_R (Fig. 4, Additional file 3: Table S2).

### Biochemistry and haematology data

Haematological, biochemical and coagulation parameters over the first 2 days are described (Additional file 3: Table S3). Serum bilirubin levels were significantly higher with ECCO_2_R compared with NIV at day 2 (14 (10–22) vs 5 (4.5–7.5) umol/L; *p* = 0.013). The platelet count was lower with ECCO_2_R compared with NIV at day 2 (96 (73–124) vs 225 (169–244) × 10^9^/L; *p* = 0.044) and baseline (96 (73–124) vs 204 (163–308) × 10^9^/L, *p* = 0.0001). Fibrinogen remained significantly higher with ECCO_2_R compared with NIV at baseline (4.3 (4.1–5) vs 2.2 (1.5–2.3) g/L, *p* < 0.001), days 1 and 2. APTTr was significantly higher with ECCO_2_R, who were on a heparin infusion at day 2 (1 (1–1.1) vs 1.6 (1.4–2.7), *p* = 0.0013).

### Complications

There were no severe or life-threatening complications in either group. The number of complications related to NIV was higher than ECCO_2_R (Additional file 3: Table S4). The majority of NIV-related complications were due to discomfort. Four patients stopped NIV due to discomfort, no patients stopped ECCO_2_R. There were no patient complications related to cannulation for ECCO_2_R. There was one ECCO_2_R cannula which thrombosed prior to commencement of ECCO_2_R and was changed without adverse incident. There was no major bleeding in either group. No patient required red blood cell transfusion. One patient with ECCO_2_R received a pool of platelets. No patient in either group underwent IMV, while they were on therapy. One patient who had received ECCO_2_R required IMV later in the hospital stay due to development of a hospital acquired pneumonia.

### Length of stay

The ICU and hospital LOS were significantly longer with ECCO_2_R than NIV (161:45 (132:27–174:50) vs 45:49 (40:22–53:00) h, *p* = 0.001 and 240:00 (219:52–337:31) vs 124:00 (103:38–213:15) h, *p* = 0.014).

### 90-day survival and symptoms at follow-up

Survival with ECCO_2_R was 6/9 (ICU), 6/9 (hospital) and 5/9 at 90-day follow-up. Survival with NIV was 9/9 (ICU), 8/9 (hospital) and 7/9 at 90-day follow-up. There was no difference in survival between NIV and ECCO_2_R at any timepoint out to 90 days (Fig. 3b, Additional file 3: Table S5).

The CAT (NIV: 22.5 (19.3–27.3), ECCO_2_R 26 (20–28)) and SGRQ (NIV: 71 (49.7–77.5), ECCO_2_R: 55.3 (54.3–64.9)) were similar at follow-up. EuroQoL 5D–5L VAS was no different (NIV: 37.5 (21.25–50), ECCO_2_R: 45 (36.25–55)).

## Discussion

The data shows that in patients with hypercapnic respiratory failure due to AECOPD, addition of ECCO_2_R to NIV leads to faster resolution of hypercapnia and tachypnoea, a significant improvement in dyspnoea and discomfort and earlier NIV discontinuation. The study demonstrates that ECCO_2_R is safe, feasible and could be commenced within 2 h of randomisation. ICU and hospital LOS were both significantly longer with ECCO_2_R.

This study has demonstrated an earlier normalisation of arterial pH with ECCO_2_R compared with NIV by more than 18 h. Given that four patients in the NIV arm withdrew from NIV against treating clinician’s advice, it is possible that this is an underestimate. The improvement in respiratory acidosis is consistent with results from observational studies exploring ECCO_2_R [11–13, 21]. In this study we report that ECCO_2_R led to a significant reduction in respiratory rate with at 8 h, while there was no reduction in respiratory rate with NIV over the first 48 h. Other studies have demonstrated a reduction in respiratory rate associated with ECCO_2_R between 1 and 24 h after commencement [11, 21]. Despite randomisation there was a difference in baseline respiratory rate between the two groups and it is possible that this contributed to the apparent improvement in respiratory rate in the first few hours after commencing ECCO_2_R.

The optimal blood flow rate for provision of ECCO_2_R is currently a subject of significant debate, with physiological evidence clearly demonstrating that higher blood flow rates are associated with greater CO_2_ clearance with a maximum sweep flow to blood flow ratio (i.e., membrane ventilation:perfusion) of 10:1 [16, 22–26]. In the present study, the blood flow was a median of 400 mL/min and the improvement in respiratory rate and acidosis suggests that in AECOPD in spontaneously breathing patients, removing CO_2_ at an average rate of ~ 90 mL/minute (roughly equivalent 30–40% of the theoretical total CO_2_ production of ~ 3 mL/kg/minute) was clinically meaningful.

Adverse consequences of NIV included significant discomfort (13/18 (72.2%) patients), consistent with other reports [4, 27]. This contributed to the withdrawal of NIV in 4/9 (44.4%) of the NIV group despite having persisting respiratory acidosis. By comparison, no patients with ECCO_2_R requested withdrawal of treatment and only one patient reported discomfort associated with the cannula insertion site.

ECCO_2_R was associated with significant and sustained improvements in dyspnoea and discomfort (Fig. 4) as measured by the VAS [28]. Dyspnoea is a complex symptom which is incompletely understood but likely relates to the neurological impact of hypoxia and hypercapnia within the brainstem as well as respiratory muscle activity [29]. Given the relationship between hypercapnia and dyspnoea, it is plausible that this resulted from the impact of ECCO_2_R on arterial CO_2_ and pH.

In keeping with other studies, adverse consequences of ECCO_2_R included development of hyperbilirubinaemia and thrombocytopaenia at day 2 [11–13, 30]. Thrombocytopaenia is commonly associated with pumped extracorporeal circuits [31]. The underlying mechanisms are incompletely understood, but may relate to platelet damage as blood transits the pump [31]. Hyperbilirubinaemia is thought to be due to red cell injury and the increase in free haemoglobin supports this [31]. Blood trauma has been linked to blood flow rates of 1L/minute or less blood flow rates, pump revolutions per minute over 3000 and negative pressures, all of which are limitations of the technique [32, 33]. Fibrinogen levels were significantly elevated with ECCO_2_R. Both hypo- and hyperfibrinogenaemia have been reported in patients requiring extracorporeal support and mechanisms are incompletely understood [30, 34]. Despite changes in platelets and fibrinogen and evidence of haemolysis there were no episodes of significant bleeding or thrombosis with ECCO_2_R and no need for blood transfusion. Other studies have shown a significant risk of bleeding with ECCO_2_R [21], possibly due to endothelial dysfunction contributed to by both the underlying disease and the circuit [35]. Other complications relating to ECCO_2_R included minor site bleeding, circuit/cannula thrombosis and one device failure and these plus the need for anticoagulation remain a limitation of the technique [30].

There is benefit for early rehabilitation in critical illness [1, 36]. It is, therefore, a little concerning that although the time to rehabilitation was did not reach statistical significance, it was numerically longer. This may be due to the route of cannulation and familiarity with mobilization with femoral cannulation. The importance of rehabilitation should be considered in future trials.

ICU and hospital lengths of stay were both 4–5 days longer with ECCO_2_R than with NIV. This was due to a longer ICU LOS—time from ICU discharge to home discharge was equal in both groups. This compares with other retrospective work which has found that the ICU LOS was shorter with ECCO_2_R compared with invasive mechanical ventilation [37]. The longer ICU stay is contributed by the differences in the protocolised care between the techniques. With NIV, nurse-led weaning occurred 24/7, based around arterial blood gases, respiratory rate and patient preference. Patients were discharged to the ward during daytime if they had been off NIV overnight. In addition, patients who consistently declined NIV (4/9) were discharged to a ward bed regardless of pH and this will have contributed to the lower ICU length of stay in the NIV arm. The protocol for patients receiving ECCO_2_R did not allow weaning overnight. There was a median of 8 h [7–24] from cessation of ECCO_2_R to decannulation and unit protocols required a further overnight stay for observation. ICU, hospital and 90-day mortality were not significantly different between groups; however, the study was not powered to detect a mortality difference. All in-hospital deaths were due to the underlying disease. Results of the CAT, SGRQ and EuroQoL 5D–5L index were not significantly different between groups and indicate that patients in both groups had significantly impaired health-related quality of life at follow-up [38].

This study is limited by the small sample size of only nine in each group, 3 patients short of the planned enrolment in each group. Despite this the primary end point of a reduction in time to cessation of NIV of at least 12 h was met. Despite randomisation there were baseline differences between groups, notably a higher respiratory rate, haemoglobin and C-reactive protein with ECCO_2_R. The withdrawal from NIV by four patients in the NIV arm led to an earlier reduction in NIV than would have been advised by the treating clinical team and resulted in earlier than expected discharge of patients from ICU at this point. These differences could have contributed to the study results and there could have been further, unmeasured differences between groups. It is possible that the higher respiratory rate at baseline led to the apparent improvement in the ECCO_2_R group. The small size limits the interpretation of the adverse consequences of ECCO_2_R as less common adverse consequences would not have been detected. Only one device was used with a 400 mL blood flow and consequently we cannot comment whether this is the optimal blood flow for management of patients with AECOPD.

The study’s strengths are that it is the first randomised, controlled trial of ECCO_2_R in a population of patients with severe COPD and severe exacerbations and powered to physiological endpoints.

## Conclusions

There is evidence of benefit associated with ECCO_2_R with time to improvement in respiratory acidosis, improvement in respiratory physiology from baseline and an immediate improvement in patient comfort and dyspnoea with commencement of ECCO_2_R and minimal clinically significant adverse events associated with its use in a population of patients with AECOPD at risk of failing or not tolerating NIV. However, the ICU and hospital lengths of stay were longer in the ECCO_2_R for similar outcomes.

## Supplementary Information

**Additional file 1 Appendix S1.** Clinical guideline.

**Additional file 2: Figure S1.** Study workflow showing the elements which impacted patient care in both arms.

**Additional file 3.** Additional tables.

## Data Availability

The data sets used and/or analysed during the current study are available from the corresponding author on reasonable request.

## Abbreviations

AECOPD: Acute exacerbations of chronic obstructive pulmonary disease
ECCO_2_R: Extracorporeal CO_2_ removal
IMV: Invasive mechanical ventilation
NIV: Non-invasive ventilation
PaCO_2_: Partial arterial pressure of carbon dioxide
