# Supplementary material for: A randomised controlled trial of non-invasive ventilation compared with extracorporeal carbon dioxide removal for acute hypercapnic exacerbations of chronic obstructive pulmonary disease
Source: Ann Intensive Care. 2022 Apr 21;12:36. doi: 10.1186/s13613-022-01006-8 (PMC9021560; doi:10.1186/s13613-022-01006-8)
Supplement: Supplementary file 1 — Additional file 1 Appendix S1. Clinical guideline. [file 13613_2022_1006_MOESM1_ESM.doc]

**Clinical Guideline**

**Veno-venous extra-corporeal CO_2_ removal (VV-ECCO_2_R)
for severe hypercapnic respiratory failure**

***Index***

***Section 1 Indications and contraindications 3***

***Section 2 Percutaneous cannulation 3***

***Section 3 Medical management of ECCO_2_R 5***

***Section 4 Nursing management of ECCO_2_R 6***

***Section 5 ECCO_2_R complications 8***

***Section 6 Audit 8***

**Introduction**

Veno-venous extracorporeal CO_2_ removal (VV ECCO_2_R) is a form of extracorporeal support where blood is removed from and returned to the venous circulation using an actively pumped system via a double lumen cannula. The difference between extracorporeal membrane oxygenation (ECMO) and ECCO_2_R is a function of the rate of blood flow. Clinically useful CO_2_ removal is possible at lower blood flows (0.5-1L) than is required for useful systemic oxygenation. ECCO_2_R is supervised by the severe respiratory failure team at GSTT.

**Section 1: Indications and contraindications for ECCO_2_R**

***Indications***

ECCO_2_R is indicated for potentially reversible, acute or acute on chronic hypercapnic respiratory failure where there is adequate oxygenation. Patients may be either on invasive or non-invasive ventilation. In line with current NICE recommendations, all patients being considered for ECCO_2_R should be offered enrolment in a trial. All patients being considered for ECCO_2_R should be discussed with the ECMO consultant prior to commencement. Pathological processes include:

- exacerbations of COPD failing non-invasive ventilation
- broncho-pleural fistula
- need for invasive mechanical ventilation following major thoracic surgery
- inability to maintain pH >7.20 due to respiratory acidosis on invasive mechanical ventilation

***Contraindications***

***Absolute contraindications to ECCO_2_R***

1. Severe (medically unsupportable) cardiovascular failure
2. Ongoing cardiac arrest
3. Advanced malignancy
4. Anaphylaxis to heparin
5. Limitations of therapy precluding ongoing critical care

**Section 2: Cannulation for ECCO_2_R**

Cannulation must be supervised by consultants who have received training in the insertion of ECCO_2_R cannulae.

***Patient preparation***

- Remove excessive hair
- Arterial line inserted (and CVC if clinically indicated)
- Prophylactic antibiotics (teicoplanin 800mg and gentamicin 5mg/kg but altered in line with known respsitance patterns and patient allergy status)
- Ensure recent coagulation studies available

***Priming***

- ¼” connections with a high flow pigtail should be cut into the circuit before and after the membrane prior to priming.
- The circuit should be primed in accordance with the manufacturer’s instructions with 1L 0.9% saline with 1 unit per mL heparin.

***Cannulation***

- Select vein for cannulation (right internal jugular or femoral veins). Note that the femoral cannula blood flow rate is lower than jugular due to its longer length.
- Full aseptic precautions
- Cannulation should be ultrasound-guided via the Seldinger technique
- The vessel should be approached at a shallow angle with the cannulation needle to ensure a straight path for the guidewire
- Ideally the skin should not be cut.
- Once wire is in place give 50 Units/kg of heparin systemically
- Serially dilate the skin and soft tissues to one size above the cannula using Coon’s dilators
- Insert cannula
- Withdraw the cannula dilator and wire in one movement. Clamp the non-metallic portion of the cannula
- Attach 20mL luer-lock syringe containing heparin-saline (10000 units in 1L 0.9% saline) to the end of each cannula, unclamp, flush and reclamp
- Following commencement of ECCO_2_R (see below), secure cannulae firmly to the skin with a minimum of 3 1.0 silk sutures.
- Dress with a biopatch and large tegaderm covering the cannulation sites, cannula-circuit connection and a portion of the circuit tubing

***Starting ECCO_2_R***

- Connect the circuit to the cannulae using a wet join
- Ensure sweep gas (air or oxygen) is connected to the device
- Remove all clamps
- Commence blood flow to maximum flow tolerated by patient, note if increase in RPM without increase in blood flow there is an increase in haemolysis. Fluid boluses may be required to improve blood flow.
- Commence CO_2_ removal, increasing sweep flow by 1L every 15 minutes and target the set pH (usually >7.30-35). A systemic blood gas as well as trans-membrane gases (pre- and post-membrane) should be measured every 15 minutes until a sweep flow of 10L/minute is reached. Trans-membrane gases assist to quantify the CO_2_ removal from the circuit and to titrate sweep gas flow rate
- Provided there are no concerns about haemorrhage (intracranial or otherwise), aim for an APTT ratio 1.5-2.0 (see ECMO Anticoagulation Protocol)

***Monitoring CO_2_ Removal***

CO_2_ removal is measured by the device. In addition, the trans-membrane blood gas should be measured across the oxygenator daily, with CO_2_ and O_2_ content difference calculated to assess membrane function.

**Section 3: Medical management of patients on ECCO_2_R**

All ECCO_2_R patients will be seen by an ECCO_2_R-trained ICU consultant at twice daily ward rounds.

***Ongoing investigations***

- - Daily ICU blood tests as per unit policy
  - APTTr 4-6 hourly as per heparin protocol
  - Daily d-dimer and fibrinogen
  - Daily LDH
  - Daily plasma free haemoglobin
  - CXR as required

***Ventilation management***

- Ventilated patients:
  - Ventilation should be protective at all times (ie <6mL/kg ideal body weight with Pplat no higher than 30cmH_2_O).
  - PEEP may need to be increased
  - Convert to spontaneous ventilation as soon as able for patients with COPD, and for patients with ARDS – maintain lung protective ventilation
- Non-ventilated patients:
  - The goals of therapy are to remove non-invasive ventilation and to replace this with high flow O_2_, titrated to an arterial saturation of 87-92%. Support should be titrated to objective and subjective work of breathing as well as aterial blood gases.
- ECCO_2_R titration. There is a non-linear removal of CO_2_ with ECCO_2_R as demonstrated below.

***Anticoagulation and blood product management***

All patients on ECCO_2_R should be systemically anticoagulated with heparin to an APTTr of 1.5-2 unless contraindicated (active bleeding, anticipated or recent surgery, thrombocytopenia). Heparin infusions are adjusted as per the guideline “Anticoagulation for the ECMO patient”.

The standard transfusion targets for the non-bleeding patient are:

- Hb 8-10
- platelets >50
- INR <1.5
- Fibrinogen >1.5

Planned procedures on ECCO_2_R will vary in their requirement for correction of clotting abnormalities and should be discussed with the consultant.

Active bleeding

- The ICU consultant must be informed of all episodes of bleeding
- Cease heparin
- Transfusion targets:
  - Platelets >80
  - INR and APTTr <1.5
  - Fibrinogen >2. Cryoprecipitate is preferred to replace fibrinogen on extracorporeal support for the bleeding patient as this also provides a rich source of von Willebrand’s Factor.
- Commence tranexamic acid as per ECMO protocol
- Source control via interventional radiology or surgery

***Weaning ECCO_2_R:***

As the underlying process improves lung compliance and work of breathing will improve. Arterial blood gases will also improve. Clinical improvement will be seen by reduction in PCO_2_ with the same minute ventilation and sweep flow. The sweep flow should be reduced to keep the pH >7.35 (adjust 2-4^th^ hourly). Once the sweep flow has been off for a period of at least 4-12 hours with respiratory rate <25 and normal pH, ECCO_2_R can be ceased and the cannulae can be removed.

***Ceasing ECCO_2_R:***

1. Cease heparin 2-4 hours prior to decannulation
2. Clamp circuit
3. Remove access cannulae using a suture to control the site (purse-string or vertical mattress). The haematocrit in the circuit and patient are the same and blood does not need to be transfused back into the patient.
4. Assess the insertion site one hour following cannula removal. Look for swelling, haematoma and adequate perfusion to the distal limb.
5. Duplex ultrasound examination vein to exclude deep vein thromboses should be performed 24-72 hours post decannulation

**Section 4: Nursing management of patients on ECCO_2_R**

All patients on ECCO_2_R require overview by a nurse with specific training in ECCO_2_R. The ECCO_2_R trained nurse is responsible for the safe co-ordination of patient care and for the safety, efficacy and troubleshooting of the ECCO_2_R circuit in collaboration with the bedside nurse. Any changes to the circuit including changes to circuit gas/blood flows and circuit anticoagulation should be discussed with the ECCO_2_R trained nurse.

The ECMO clinical nurse specialists provide a 24-hour reference for all patients on ECCO_2_R.

***Patient Management***

In addition to routine patient care as per unit policy;

- 4-6hrly APTTr
- Titration of heparin infusion according to specified APTTr target
- 12hrly vascular observations in the cannulated limb
- Maintain the safety of the ECCO_2_R circuit during any patient movement (rolls, CXRs, procedures, pressure area care) to ensure that no tension is transmitted to the cannula and that the circuit tubing is not kinked.
- Use Rusch suction catheters for oral suction (not yankeur suckers)
- Use green mouth swabs for any mouth care (not toothbrushes)
- Review bloods daily to observe for any signs of haemolysis/DIC (Hb, platelets, fibrinogen, D-Dimers) and monitor urine carefully for any signs of haematuria

***Circuit Management***

- Safety checks performed on commencement of shift to include:
  - ECCO_2_R plugged into UPS plug
  - Pump auscultation
  - Blood pump flow rate
  - Sweep flow rate
  - Membrane gas exchanger inspection
  - Check patient’s position and insertion sites
  - Check pulses and peripheral perfusion in the cannulated limb
  - Pump access line movement (shaking, swinging, still)
  - 2 sets of circuit clamps available
  - Record gas source (air or oxygen)
  - Change sweep gas canister every 24 hours (ECCO_2_R trained nurse)
  - Ensure seal flush is connected and recorded on the fluid balance chart (ECCO_2_R nurse to replace infusion as required)
  - Ensure circuit is secured to the bed
- Circuit observations
  - Record observations hourly in the ventilator section of the ICU flowsheet;
    - RPMs
    - Blood flow
    - Sweep gas flow
    - Sweep gas type (air/oxygen)
    - CO_2_ removal mls/min
    - Insertion site bleeding (yes/no)
    - ECCO_2_R complications (drop down list)
- Blood gases
  - Daily simultaneous ABG and pre and post-membrane blood gases.
    - Ensure the ctCO_2_(B) from each blood gas is recorded in the blood gases flowsheet
    - Note the calculated CO_2_ extraction in the Vent Obs flowsheet
    - Manually enter the VCO_2_ from the ECCO_2_R device into the correct row in the Vent Obs flowsheet

**Section 5: Complications with ECCO_2_R**

***Haemolysis***

Haemolysis, defined by a plasma free haemoglobin of >0.5g/dL, is the commonest complication associated with ECCO_2_R. It is usually caused by having too high a RPM for the blood flow. To avoid the development of haemolysis, stop increasing RPM if there is no additional blood flow associated. If haemolysis develops, reduce RPM. Rarely haemolysis is due to thrombus forming within the centrifugal pump in which case, the circuit will need to be changed.

***Low Flow***

The low flow alarm is triggered by having a circuit blood flow of less than 300mL/min. The causes include hypovolaema, cannula/tubing kinking and changes in patient position. The line and patient should be assessed if the low flow alarm occurs. Immediate responses include volume resuscitation and line manipulation.

***Decannulation***Decannulation is a preventable problem. The line site should be inspected on each shift to document line position. Line sites should also be inspected following patient moves including mobilisation and trips to CT/theatres. The sutures should be inspected at the same time to ensure they are fixing the cannula appropriately. Any changes in position should be reported to the consultant.

### ***Circuit Air Embolism*** If this occurs, clamp and cease the circuit, inform the consultant, ensure that the source of air is identified and controlled and prime a new circuit to continue ECCO_2_R (if required).

### ***Gas exchange membrane failure*** This is shown by a progressive reduction in gas exchange across the membrane. If this occurs, the circuit needs to be changed.

**Section 6: Audit**

The medical and nursing leads for ECMO will audit all aspects of the ECCO_2_R service and will report cases to ELSO (the extracorporeal life support organisation). Any national reporting required by NICE or other appropriate organisation will also be undertaken.
