# Supplementary figures and images for: A randomised controlled trial of non-invasive ventilation compared with extracorporeal carbon dioxide removal for acute hypercapnic exacerbations of chronic obstructive pulmonary disease
Source: Ann Intensive Care. 2022 Apr 21;12:36. doi: 10.1186/s13613-022-01006-8 (PMC9021560; doi:10.1186/s13613-022-01006-8)

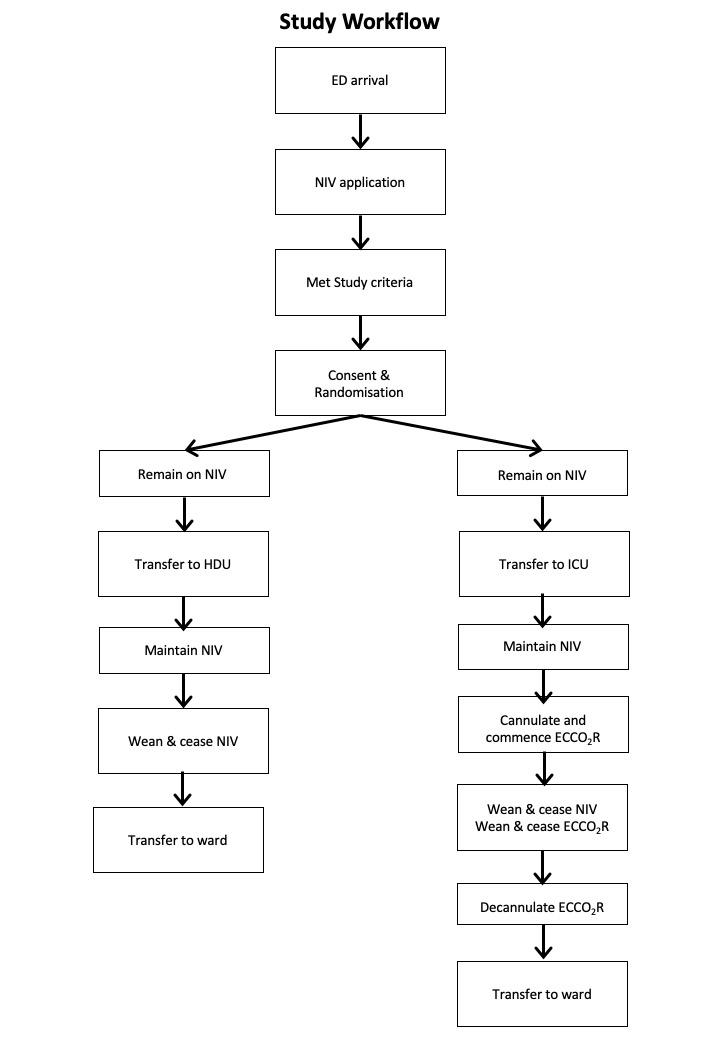

Supplement: Supplementary file 2 — Additional file 2: Figure S1. Study workflow showing the elements which impacted patient care in both arms. [file 13613_2022_1006_MOESM2_ESM.jpeg]
