# Supplementary material for: A randomised controlled trial of non-invasive ventilation compared with extracorporeal carbon dioxide removal for acute hypercapnic exacerbations of chronic obstructive pulmonary disease
Source: Ann Intensive Care. 2022 Apr 21;12:36. doi: 10.1186/s13613-022-01006-8 (PMC9021560; doi:10.1186/s13613-022-01006-8)
Supplement: Supplementary file 3 — Additional file 3. Additional tables. [file 13613_2022_1006_MOESM3_ESM.docx]

Additional file 1: Table S1: NIV observations, basic physiological data and arterial blood gases over the first 48 hours (median (IQR)). * p<0.05 difference between NIV and ECCO_2_R; § p<0.05 difference between measurement and baseline within in the ECCO_2_R group; # p<0.05 difference between measurement and baseline within in the NIV group

|  | **NIV** | | | | | |  |
| --- | --- | --- | --- | --- | --- | --- | --- |
|  | **Baseline** | **4 hours** | **8 hours** | **12 hours** | **24 hours** | **48 hours** |  |
| **NIV Observations** |  |  |  |  |  |  |  |
| EPAP (cmH_2_O) | 5 (5-5) | 5 (5-5.25) | 5 (5-6.5) | 5.5 (5-6.5) | 7 (5-8) | 7.5 (6.5-8) |  |
| IPAP (cmH_2_O) | 18 (15-22) | 17 (11.5-22.75) | 18.5 (12-22) | 20.5 (12-22) | 19 (12-22) | 20.5 (17.25-22.5) |  |
| FiO2 (%) | 32 (26-40) | 28 (26-40) | 30 (28-32) | 30 (28-32) | 28 (24-28) | 28 (27-28.5) |  |
| **Physiological data** |  |  |  |  |  |  |  |
| RR (breaths/min) | 24 (20-28)* | 20 (19-24) | 19 (17-22) | 17 (15-19) | 17 (15-22) | 20.5 (20-22.75) |  |
| SpO_2_ (%) | 90 (89-92) | 90 (89-92) | 91 (90-92) | 92 (90-94) | 93 (91-94) | 94.5 (93.75-96) |  |
| HR (beats/min) | 100 (90-105) | 99 (99-105) | 98 (91-104) | 93 (87-101) | 96 (91-99) | 88 (82.5-93.75) |  |
| SBP (mmHg) | 120 (105-144) | 123 (119-137) | 132 (117-138) | 137 (114-138) | 128 (115-145) | 118 (109-130) |  |
| **Arterial blood gas** |  |  |  |  |  |  |  |
| PaO_2_ (kPa) | 8.37 (8.05-9.35) | 8.77 (7.74-8.95) | 8.84 (7.93-10.15) | 8.2 (8.01-8.6) | 7.81 (7.55-8.14) | 8.12 (7.54-8.68) |  |
| pH | 7.27 (7.21-7.27) | 7.3 (7.26-7.33) | 7.32 (7.28-7.33)# | 7.34 (7.31-7.35)# | 7.36 (7.33-7.38)# | 7.38 (7.36-7.40) |  |
| PaCO_2_ (kPa) | 9.16 (8.23-10.02) | 8.3 (7.74-9.3)* | 8.18 (7.63-8.7) | 8.35 (7.6-9.45) | 7.51 (7.16-8.92) | 7.40 (7.16-8.08) |  |
| HCO_3_ (mmol/L) | 29.1 (26.7-33.6) | 25.5 (22.2-30.4) | 26.5 (24.2-29.2) | 26.4 (25.5-31) | 27.3 (26.1-30) | 29.4 (29-30.175) |  |
|  | **ECCO_2_R** | | | | | |  |
|  | **Baseline** | **4 hours** | **8 hours** | **12 hours** | **24 hours** | **48 hours** | |
| **NIV Observations** |  |  |  |  |  |  | |
| EPAP (cmH_2_O) | 6 (5-6) | 5 (5-6) | 5 (4.5-5) | 5 (5-5) |  |  | |
| IPAP (cmH_2_O) | 18 (16-20) | 18 (16-20) | 18 (14.5-20.5) | 20 (20-20) |  |  | |
| FiO2 (%) | 35 (28-40) | 35 (30-40) | 35 (28-40) | 30 (28-40) | 30 (25-40) | 35 (35-40) | |
| **Physiological data** |  |  |  |  |  |  | |
| RR (breaths/min) | 29 (26-32)* | 22 (20-25)§ | 20 (20-22)§ | 22 (20-24)*§ | 21 (20-23)§ | 17 (16-23)§ | |
| SpO_2_ (%) | 91 (87-93) | 92 (88-96) | 92 (91-94) | 90 (86-91) | 92 (90-93) | 92 (91-95) | |
| HR (beats/min) | 101 (100-105) | 101 (97-108) | 93 (90-99) | 87 (83-106) | 88 (88-102) | 92 (84-101) | |
| SBP (mmHg) | 130 (112-139) | 142 (127-158) | 141 (130-158) | 123 (115-158) | 123 (115-149) | 119 (115-147) | |
| **Arterial blood gas** |  |  |  |  |  |  | |
| PaO_2_ (kPa) | 8.89 (7.9-9.49) | 7.77 (7-8.9) | 8.45 (7.6-8.78) | 7.67 (7.21-8.9) | 9.02 (8.19-9.52) | 8.8 (8.48-8.9) | |
| pH | 7.27 (7.25-7.29) | 7.35 (7.31-7.37)§ | 7.32 (7.32-7.38)§ | 7.37 (7.35-7.41)§ | 7.37 (7.33-7.39)§ | 7.39 (7.37-7.42)§ | |
| PaCO_2_ (kPa) | 9.34 (8.49-10.2) | 6.8 (6.2-7.15)*§ | 8.13 (7.02-8.2)§ | 7.99 (7.05-8.38)§ | 7.51 (6.88-8.09)§ | 8.02 (6.57-8.3)§ | |
| HCO_3_ (mmol/L) | 27.9 (26.9-35.5) | 24.7 (23.2-27.8) | 25.7 (25-29) | 26.5 (25.1-28.4) | 26.6 (25.1-30.8) | 29.7 (27.8-31.4) | |

Additional file 1: Table S2: Visual analogue scores for the two groups (median (IQR)). * p<0.05 difference between NIV and ECCO_2_R; § p<0.05 difference between measurement and baseline within in the ECCO_2_R group; # p<0.05 difference between measurement and baseline within in the NIV group

|  | Pre-ECCO_2_R | Day 1 | Day 2 |
| --- | --- | --- | --- |
| **NIV** |  |  |  |
| Discomfort |  | 56 (37-87) | 50 (33-89) |
| Dyspnoea |  | 41 (28-68) | 24 (18-46) |
| **ECCO2R** |  |  |  |
| Discomfort | 84 (78-87) | 13 (4-65)§ | 8 (1-67)§ |
| Dyspnoea | 85 (80-87) | 20 (7-52)§ | 7 (2-28)§ |

Additional file 1: Table S3: Haematological, biochemical and coagulation parameters (median (IQR)), * p<0.05 difference between NIV and ECCO_2_R; § p<0.05 difference between measurement and baseline within in the ECCO_2_R group; # p<0.05 difference between measurement and baseline within in the NIV group

|  | **NIV** | | | **ECCO_2_R** | | |
| --- | --- | --- | --- | --- | --- | --- |
| **Day** | **Baseline** | **Day 1** | **Day 2** | **Baseline** | **Day 1** | **Day 2** |
| **Renal Biochemistry** |  |  |  |  |  |  |
| Sodium (mmol/L) | 138 (132-141) | 139 (135-141) | 140 (139-141.5) | 139 (138-142) | 141 (140-143) | 142 (142-143) |
| Potassium (mmol/L) | 4.8 (4.6-5) | 4.5 (4.2-4.6) | 3.8 (3.7-4.4) | 4.2 (3.8-4.5) | 4.5 (4.3-4.5) | 4.4 (4-5) |
| Creatinine (umol/L) | 99 (57-136) | 68.5 (52.5-92.25) | 67 (55.5-93) | 77 (69-80) | 58 (49-76) | 58 (51-74) |
| free Hb (g/dL) |  |  |  | 0 (0-0.1) | 0.4 (0.2-0.5)§ | 0.3 (0.3-0.6)§ |
| **Hepatic function** |  |  |  |  |  |  |
| Alanine transaminase (IU/L) | 20 (17-24) | 22 (16.75-24.5) | 21 (15-22.75) | 19 (15-33) | 24 (15-29) | 22 (18-36) |
| Total bilirubin (umol/L) | 6 (4-6) | 7 (4-9.25) | 5 (4.5-7.5) | 7 (5.5-12) | 14 (8-26) | 14 (10-22)*§ |
| **Inflammatory markers** |  |  |  |  |  |  |
| C-reactive protein (mg/L) | 13 (4-21) | 12 (5-64) | 7 (3.25-66) | 32 (30-51)* | 47 (20-56) | 28 (23-41) |
| **Haematology** |  |  |  |  |  |  |
| Leukocytes (x10^9^/L) | 8.9 (6.8-10.4) | 9.2 (8.7-10.3) | 9.6 (8.35-10.35) | 9.1 (8.3-11.8) | 9.1 (6.9-11.7) | 8.5 (6.3-13.8) |
| Haemoglobin (g/L) | 130 (120-136) | 116 (107-126) | 108 (100-117) | 151 (143-157)* | 126 (113-127) | 115 (105-125) |
| Platelets (x10^9^/L) | 251 (172-288) | 224 (195.5-229.75) | 225 (169-244) | 204 (163-308) | 138 (113-176)§ | 96 (73-124)*§ |
| **Coagulation** |  |  |  |  |  |  |
| INR | 1 (0.9-1.1) | 1 (0.9-1.125) | 1 (1-1.1) | 1 (1-1.1) | 1 (1-1.1) | 1 (1-1) |
| Fibrinogen (g/L) | 2.2 (1.5-2.3) | 1.85 (1.45-2.4) | 2 (1.75-2.6) | 4.3 (4.1-5)* | 3.8 (3.4-4.4)* | 3.7 (3.2-4.7)* |

Additional file 1: Table S4: Adverse events relating to NIV and ECCO_2_R.

| **NIV (n=18)** | **n** | **ECCO_2_R (n=9)** | **n** |
| --- | --- | --- | --- |
| NIV failure | 0 | Device failure | 1 |
| Aspiration pneumonia | 0 | Circuit change | 0 |
| Barotrauma | 0 | Cannula site bleeding | 3 |
| Hypotension (SBP <80mmHg) | 0 | Cannula site infection - clinical | 0 |
| Arm oedema | 0 | Cannula site infection – microbiological | 0 |
| CO_2_ rebreathing | 0 | Air embolus | 0 |
| Claustrophobia | 1 | Haemolysis | 3 |
| Discomfort (patient reported) | 13 | Discomfort (patient reported) | 1 |
| Mechanical | 1 | Line position change | 0 |
| Nasal skin lesions | 1 | Circuit thrombus | 0 |
| Air leaks | 8 | Circuit fracture | 0 |
| Airway dryness | 2 | Intracranial haemorrhage | 0 |
| Gastric insufflation | 0 | Haemorrhage requiring 2 or more units | 0 |
| Vomiting | 1 | Venous insufficiency | 0 |
|  |  |  |  |
| Tracheal intubation required | 0 | Tracheal intubation required | 1 |
| Withdrew from therapy | 4 | Withdrew from therapy | 0 |
| Deep vein thrombosis | 0 | Deep vein thrombosis | 0 |

Additional file 1: Table S5: Outcomes at ICU discharge, hospital discharge and 90 days.

|  | **NIV** | **ECCO_2_R** |
| --- | --- | --- |
| ICU survival | 9/9 (100%) | 6/9 (66%) |
| Hospital survival | 8/9 (89%) | 6/9 (66%) |
| 90-day survival | 7/9 (78%) | 5/9 (56%) |
| COPD assessment test | 22.5 (19.3-27.3) | 26 (20-28) |
| EuroQol 5D-5L VAS | 37.5 (21.25-50) | 45 (36.25-55) |
| St George's respiratory questionnaire | 71 (49.7-77.5) | 55.3 (54.3-64.9) |

| **Hours** | **4** | **8** | **12** | **24** | **48** |
| --- | --- | --- | --- | --- | --- |
| **Pump RPM** | 1400 (1400-1400) | 1400 (1400-1400) | 1400 (1400-1400) | 1400 (1400-1400) | 1400 (1400-1400) |
| **Blood flow (mL/min)** | 370 (357.5-380) | 380 (380-395) | 385 (377.5-400) | 400 (365-410) | 410 (395-420) |
| **Sweep flow (L/min)** | 5 (3.5-8.5) | 10 (6.5-10) | 10 (7.5-10) | 10 (10-10) | 10 (7-10) |
| **VCO_2_ (mL/min)** | 78.5 (71.25-96.25) | 88 (82.25-110.25) | 90 (76.75-109.5) | 95 (81.5-97) | 86 (72.25-101.25) |

Additional file 1: Table S6: ECCO_2_R settings for the first 48 hours. All are median (IQR). VCO_2_ is the reported membrane CO_2_ clearance by the device.
